# Comorbidity between Borderline Personality Disorder, Eating Disorders and Suicide Risk: a Narrative Literature Review

**DOI:** 10.1192/j.eurpsy.2026.11472

**Published:** 2026-07-31

**Authors:** P. F. B. Araújo, V. L. de-Melo-Neto, A. F. C. Ximenes, L. V. de Araújo, B. D. A. A. L. T. de Melo, A. B. D. C. Alves, L. C. Carneiro, M. I. O. de Morais, F. O. Barbosa, E. D. F. Correia, D. B. B. Ferreira

**Affiliations:** 1 Psychiatry, Universidade de Ciências da Saúde de Alagoas; 2 Psychiatry, Universidade Federal de Alagoas, Maceió, Brazil

## Abstract

**Introduction:**

Borderline personality disorder (BPD) and eating disorders represent two of the most severe psychiatric conditions associated with elevated suicide risk. When these conditions co-occur, clinical complexity increases substantially, amplifying suicidal behaviors and mortality rates. Understanding suicide risk in this group is crucial for developing effective prevention strategies and improving clinical outcomes.

**Objectives:**

This narrative literature review examines suicide risk in patients with comorbid borderline personality disorder and eating disorders, identifying risk factors, prevalence rates, and clinical implications to inform evidence-based prevention and treatment approaches.

**Methods:**

This narrative literature review was conducted using PubMed, Embase, and PsychINFO databases from 2020-2025. Search terms included “borderline personality disorder,” “eating disorders,” “suicide,” and “comorbidity.” Studies were eligible if they: (1) examined suicide rates or risk factors, (2) included patients with comorbid BPD and eating disorders, and (3) were published in English or Portuguese.

**Results:**

Research demonstrates significantly elevated suicide risk in patients with comorbid BPD and eating disorders compared to either condition alone.

Studies indicate that 28.75% of individuals with BPD have attempted suicide, with comorbid eating disorders further increasing this risk.

Treatment-seeking populations with BPD and comorbid eating disorders show particularly high rates of self-harm and suicidal behaviors.

Contemporary research reveals that eating disorders, particularly bulimia nervosa, serve as independent risk factors for suicide regardless of psychiatric comorbidities. The combination of BPD and eating disorders creates a synergistic effect on suicide risk, driven by shared features such as impulsivity, emotional dysregulation, and self-destructive behaviors. Furthermore, the use of immature defense mechanisms, a common trait in BPD, significantly predicts suicide attempts in this population. Treatment complexity increases substantially in this population, requiring specialized integrated approaches addressing both conditions simultaneously. Several well-known mechanisms underlying BPD are also common in eating disorders, manifesting in both symptoms and interpersonal difficulties. These include poor awareness of affect, alexithymia, and perfectionism.

**Conclusions:**

Patients with comorbid borderline personality disorder and eating disorders face substantially elevated suicide risk compared to either condition alone. The combination creates a synergistic effect on suicidal behaviors, requiring specialized assessment and intervention strategies. Early identification, comprehensive risk assessment, and integrated treatment approaches are essential for suicide prevention. Future research should focus on developing specific risk assessment tools and evidence-based interventions.

**Disclosure of Interest:**

None Declared

